# Correlation between fasting blood glucose level and risk of breast cancer in women: a single-center, prospective cohort study

**DOI:** 10.3389/fonc.2024.1359839

**Published:** 2024-07-01

**Authors:** Gefei Li, Mingjie Yin, Zhimin Fan, Fengjiang Qu

**Affiliations:** ^1^ Department of Breast Surgery, General Surgery Center, The First Hospital of Jilin University, Xinmin, Changchun, Jilin, China; ^2^ Tangshan Workers’ Hospital Affiliated to Hebei Medical University, Wenhua, Tangshan, Hebei, China; ^3^ Department of Emergency Surgery, The First Hospital of Jilin University, Xinmin, Changchun, Jilin, China

**Keywords:** breast cancer, fasting blood glucose, TyG index, prospective cohort, young breast cancer

## Abstract

**Purpose:**

We prospectively analyzed the correlation between fasting plasma glucose (FPG) and the risk of breast cancer in women; explored the independent risk factors for breast cancer in women, and compared the effect of FPG level on the risk of young and non-young breast cancer. Our study provides new evidence and ideas for research into breast cancer etiology in China, improves the accuracy of secondary prevention of breast cancer, and provides options for the clinical diagnosis and treatment of breast cancer patients with diabetes.

**Materials and methods:**

Three cohorts of women participating in the first health examination of the Kailuan Group in 2006, 2008 and 2010 were assembled to conduct a descriptive analysis of the baseline data on FPG. The cumulative incidence of breast cancer in different groups over 13 years was calculated using the Kaplan-Meier method and groups were compared using the log-rank test. A Cox proportional hazards regression model was used to analyze the association between FPG level and the risk of breast cancer.

**Results:**

The cumulative incidence of breast cancer increased in people with FPG higher than 5.29 mmol/L, but there was no significant difference in the effect of different levels of FPG on the risk of young breast cancer in the population. Different degrees of fasting glucose can affect the risk of non-young breast cancer in the population.

**Conclusion:**

The results of this study suggest that the risk of breast cancer can be reversed by early intervention to control levels of FPG. Regular monitoring of FPG may reduce the misdiagnosis rate of breast cancer in the population.

## 1 Background

As the “first killer” of women’s physical health, the risk factors for the occurrence and development of breast cancer have always held the attention of investigators. The occurrence of malignant tumors in women is the result of various risk factors, one of which is elevated fasting blood glucose (fasting plasma glucose, FPG), which has been found to be a high-risk factor for the occurrence and development of malignant tumors (1–4). There is a linear correlation between elevated FPG levels and the risk of breast cancer (5–7). In addition, high levels of FPG also increase the risk of postoperative recurrence (8) and distant metastasis in women (9), and they significantly affect the efficacy of postoperative chemotherapy in breast cancer patients (10). Meanwhile, a high level of the triglyceride-glucose index (TyG) is an independent risk factor for type 2 diabetes, and indirectly may also be one of the potential risk factors for breast cancer. At present, research concerning FPG and the cumulative incidence of female breast cancer is still incomplete, as there are few comparative studies on the cumulative incidence of young and non-young breast cancer; furthermore, the conclusions of these studies are not consistent, requiring further data support. The present study aims to prospectively explore the cumulative risk for breast cancer caused by elevated FPG, covering a follow-up period of 13 years. Moreover, we sought to determine if controlling blood sugar may help prevent breast cancer and lower recurrence and metastasis measures (11, 12). We further examined whether timely detection of FPG and regular physical examinations can improve the detection rate of female breast cancer and increase secondary prevention of breast cancer. Finally, we studied the relationship between the diabetes and breast cancer treatment, which may provide perspective for the clinical diagnosis and treatment of diabetic breast cancer patients.

## 2 Research method

### 2.1 Study participants and subgroups

**Figure 1:**
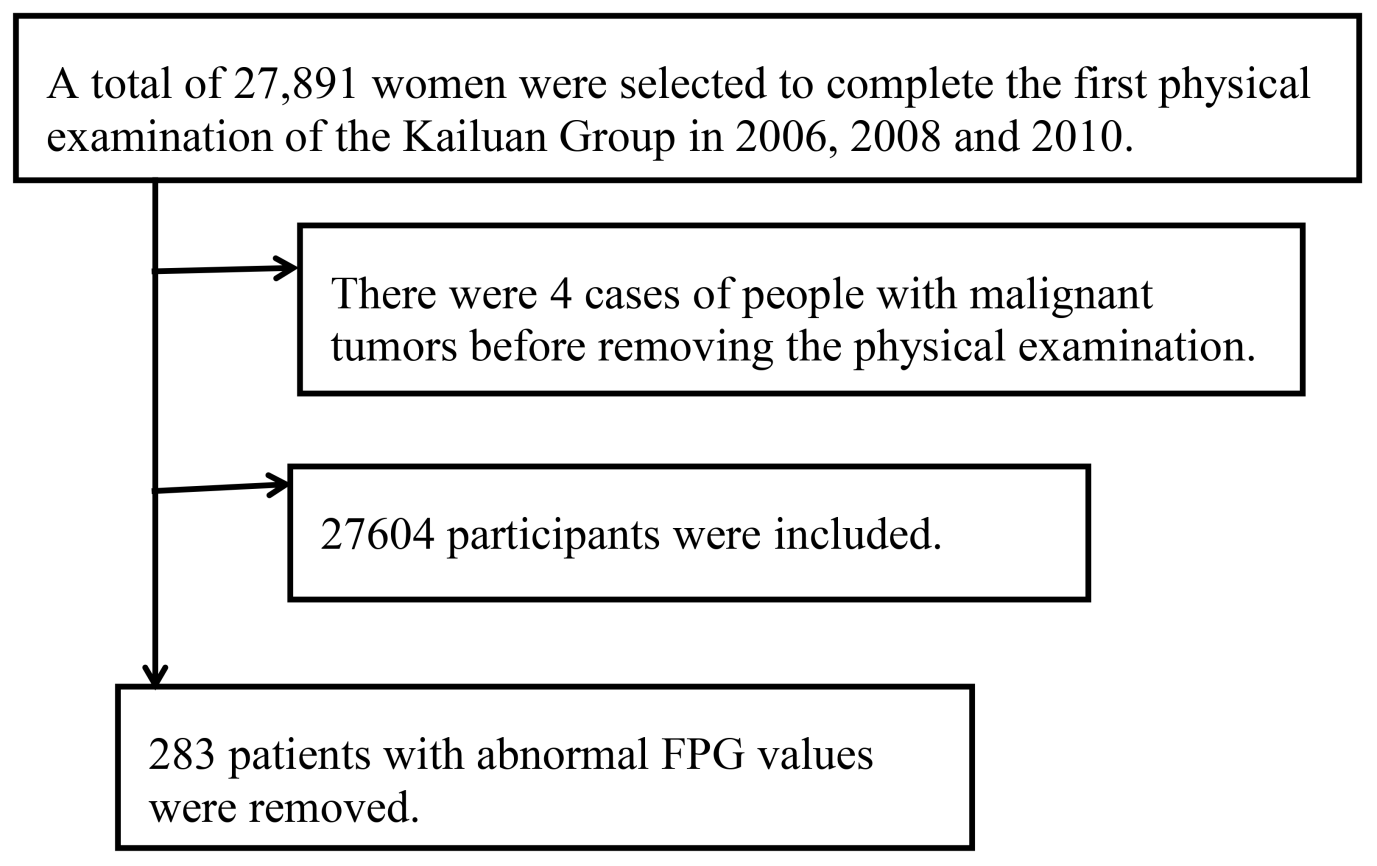
Inclusion and exclusion flowchart.

We selected women who participated in the first physical examination of the Kailuan Group in 2006, 2008 and 2010. Patients with abnormal FPG caused by various factors were excluded (see **Figure 1**).

Based on the large number of single-center clinical studies at home and abroad, the participants were divided into three groups according to FPG levels: Q1 (fasting glucose <4.72 mmol/L); Q2 (fasting glucose 4.72–5.29 mmol/L) and Q3 (fasting glucose >5.29 mmol/L). In addition, based on the TyG index, the participants were also divided into three groups: W1 group: TyG index <8.17 mg/dl; W2 group: TyG index 8.17 mg/dl–8.70 mg/dl; and W3 group: TyG index >8.70 mg/dl.

### 2.2 Data collection

Epidemiological data collected included demographic data (age, gender), health behavioral habits (smoking, alcohol consumption, physical exercise, salt intake), anthropometric indicators (height, weight, systolic and diastolic blood pressure), and blood biochemical indicators, including FPG, triglycerides, total cholesterol, high-density lipoprotein cholesterol, and low-density lipoprotein cholesterol.

### 2.3 Relevant definitions

TyG index = Ln (fasting blood glucose * triglycerides/2) in mg/dl. Waist-to-hip ratio = waist/hip circumference. Smoking: average numbers of cigarettes per day in the previous year. Alcohol consumption: in the previous 1 year, women drank on average one standard alcohol drink/day (consisting of 100 g beer alcohol, 5.0 g alcohol; 100 g wine alcohol, 12.0 g; 100 g liquor alcohol, 40.0 g, 1 standard alcohol 14.0 g). Regular physical exercise was defined as exercise three times per week for at least 30 min duration. Salt intake: light daily intake of sodium chloride, <6 g; normal daily intake of sodium chloride, 6–10 g; heavy daily intake of sodium chloride, >10 g. Young breast cancer: breast cancer with an age of onset <35 years of age; non-young breast cancer: ≥35 years (13).

### 2.4 Follow-up starting point and endpoint events

The completion of the first health examination of the Kailuan Group in 2006, 2008 or 2010 was adopted as the starting point of the follow-up, while the endpoint event was breast cancer occurring during the follow-up period. The definition and diagnostic criteria were adopted from the World Health Organization criteria, namely, the National Comprehensive Cancer Network guidelines. The time of the starting point event and endpoint event were recorded, with the endpoint event occurring no later than December 31, 2020. If the participant did not have an endpoint event but died during the follow-up period, the time of death was considered the end of follow-up.

### 2.5 Statistical methods

For descriptive analysis of baseline data of study participants, normal measurement data were expressed as means ± standard deviation (x ± s) for variance analysis; as medians (M [P25, P75]) for Kruskal-Wallis rank-sum tests for group comparisons; count data were expressed by n (%); and comparisons between groups (2 test). Missing values of covariates were filled by multiple interpolation.

The cumulative incidence of breast cancer in different groups was calculated using the Kaplan-Meier method and compared using the log-rank test. The Cox proportional hazards regression model was used to analyze the association of FPG levels with the risk of breast cancer disease. To test the stability of the results, we performed a sensitivity analysis, using the Cox proportional hazards regression model to analyze the role of the TyG index on the risk of breast cancer disease. Two-sided tests at P < 0.05 were considered to indicate statistically significant differences.

## 3 Results

### 3.1 Comparison of baseline data on breast cancer disease in different groups

In the study population of 27,604 women, the mean age was 47.53 ± 11.95 years. Compared with the Q1 group, age, triglycerides, total cholesterol, LDL cholesterol, systolic blood pressure, diastolic blood pressure, alcohol consumption and the smoking rate were higher in the Q2 group, while measures of HDL cholesterol, physical exercise frequency and salt intake was lower in the Q2 vs Q1 group. Compared with the Q1 group, age, triglycerides, total cholesterol, LDL cholesterol, systolic BP, diastolic blood pressure, smoking rate, and physical exercise frequency were higher in the Q3 group, while measures of HDL cholesterol, alcohol consumption and salt intake were lower (see **Table 1** for details).

**Table 1:** Baseline data (N = 27,604).

Variable	Q1	Q2	Q3	F/χ^2^ rate	P rate
Age ( $\bar{\text{x}}$ ± s, year)	45.82 ± 12.53	45.99 ± 11.96	50.74 ± 11.35	503.62	<0.01
Waist-to-hipratio ( $\bar{\text{x}}$ ± s)	0.86 ± 0.08	0.86 ± 0.08	0.87 ± 0.08	132.95	<0.01
Fasting blood-glucose ( $\bar{\text{x}}$ ± s, mmol/L)	4.32 ± 0.33	4.99 ± 0.16	6.57 ± 2.14	7,818.21	<0.01
Glycerin trilaurate [M (P_25_, P_75_), mmol/L]	1.03 (0.72, 1.50)	1.07 (0.75, 1.55)	1.34 (0.93, 1.99)	1,097.63	<0.01
Cholesterol total ( $\bar{\text{x}}$ ± s, mmol/L)	4.78 ± 1.04	4.87 ± 1.02	5.12 ± 1.14	247.02	<0.01
High density lipoprotein cholesterol ( $\bar{\text{x}}$ ± s, mmol/L)	1.59 ± 0.47	1.55 ± 0.38	1.57 ± 0.43	15.76	<0.01
Low density lipoprotein cholesterol [M (P_25_, P_75_), mmol/L]	2.09 (1.60, 2.65)	2.23 (1.74, 2.78)	2.44 (1.92, 3.02)	816.88	<0.01
Systolic pressure ( $\bar{\text{x}}$ ± s, mmHg)	119.16 ± 19.79	120.66 ± 19.47	129.00 ± 21.76	624.69	<0.01
Diastolic pressure ( $\bar{\text{x}}$ ± s, mmHg)	77.16 ± 10.78	78.01 ± 10.65	81.56 ± 11.13	426.72	<0.01
Smoke [n (%)]	172 (1.89)	158 (1.71)	192 (2.07)	3.27	<0.01
Drink [n (%)]	601 (6.61)	630 (6.82)	517 (5.58)	13.75	<0.01
Do physical exercise regularly [n (%)]	1,199 (13.19)	1,048 (11.34)	1,407 (15.18)	59.44	<0.01
Salt habit the taste is light [n (%)]	1,036 (11.40)	974 (10.54)	1,012 (10.92)	10.33	<0.01

### 3.2 Cumulative incidence and incidence density of breast cancer disease in different groups

**Figure 2:**
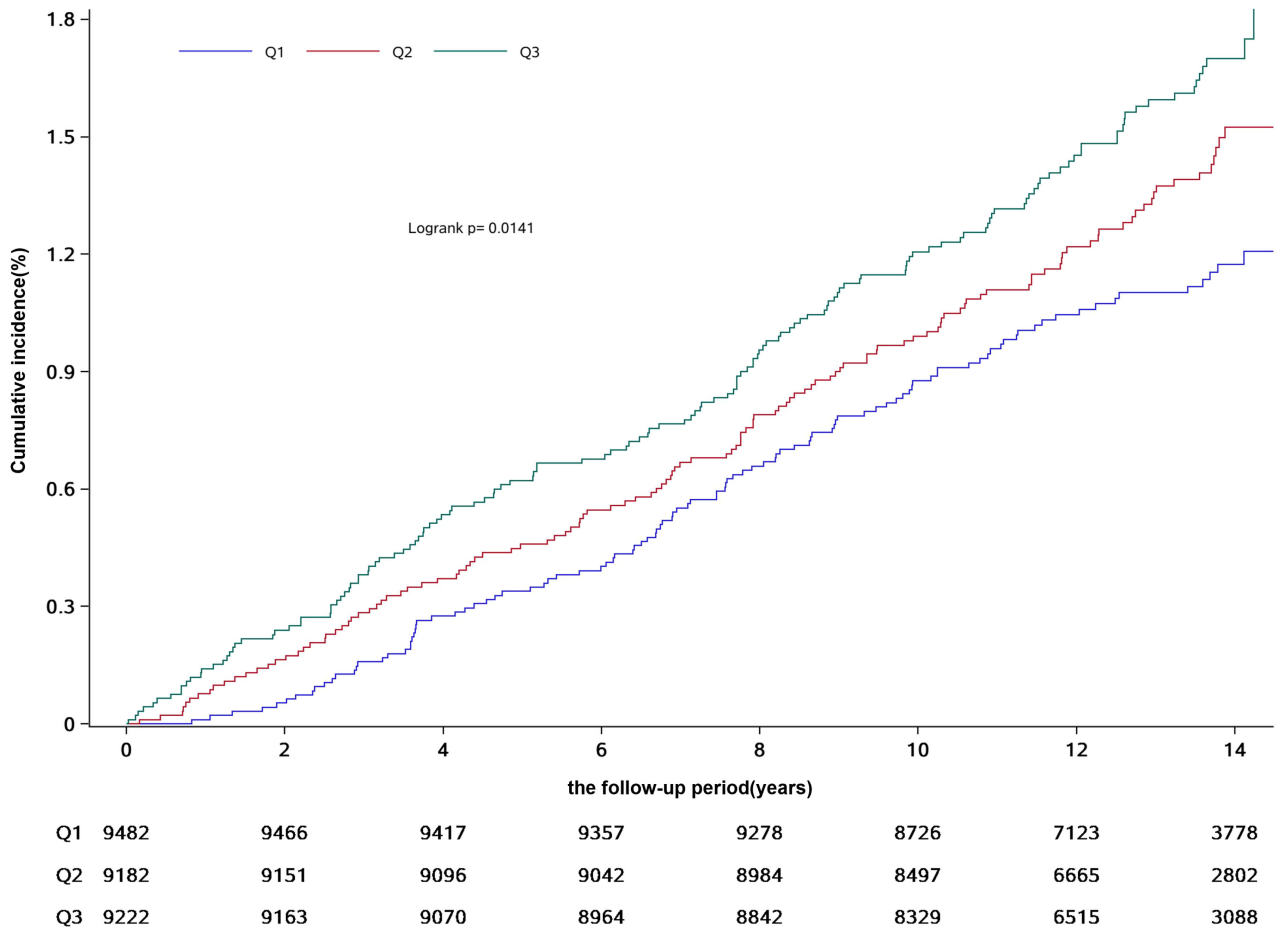
Cumulative incidence of breast cancer in participants from different groups.

During the mean follow-up period of 12.90 ± 2.03 years, 375 of the participants included in the study developed breast cancer during the follow-up period. The cumulative incidence of breast cancer in the three groups was 1.20% (103/9,091), 1.53% (126/9,243) and 1.70% (146/9,270) in groups Q1, Q2 and Q3, respectively, (χ2 = 7.65, P = 0.02, see **Table 2** and **Figure 2**). The incidence density was 0.87/thousand, 1.06/thousand and 1.24/thousand in groups Q1, Q2 and Q3, respectively (see **Table 2** for details).

**Table 2:** Cumulative incidence and incidence density of breast cancer disease among different participant groups.

Groups	Number of incident cases/total number	Disease onset density (/thousand person-years)	Cumulative incidence rate (%)	P rate
Breast cancer disease				
Q1	103/9,091	0.87	1.20	0.01
Q2	126/9,243	1.06	1.53	
Q3	146/9,270	1.24	1.70	

### 3.3 Multivariate Cox regression analysis of the effect of participant groups on disease risk of breast cancer

In the entire population, the Q2 and Q3 groups had hazard ratios (HR) of 1.21 (95% confidence interval [CI]: 0.93–1.57) and 1.30 (95% CI: 1.00–1.68; (see **Table 3** for details).

**Table 3:** Multivariate Cox regression analysis of the effect of FPG on the risk of breast cancer.

Groups	Model 1		Model 2	
	HR rate (95% CI)	P rate	HR rate (95% CI)	P rate
The whole crowd (n = 27,604)				
Triple tile of fasting blood glucose				
Q1	1		1	
Q2	1.22 (0.94, 1.58)	0.14	1.21 (0.93, 1.57)	0.15
Q3	1.32 (1.03, 1.71)	0.03	1.30 (1.00, 1.68)	0.05
	P for trend = 0.03		P for trend = 0.05	
For every 1 mmol/L increase in fasting blood glucose	1.03 (0.98, 1.09)	0.26	1.03 (0.97, 1.09)	0.40
For every 1 standard deviation increase in fasting blood glucose^a^	1.05 (0.96, 1.15)	0.26	1.04 (0.95, 1.14)	0.40

Model 1 corrected for age; model 2 corrected for systolic blood pressure, waist-hip ratio <0.8 or waist-hip ratio = 0.8), triglycerides, total cholesterol, frequency of physical exercise (infrequent or frequent), smoking (yes or no), alcohol consumption (yes or no), and salt intake (light or moderate). ^a^1.57.

### 3.4 Multivariate Cox regression analysis of the effect of FPG on disease risk of young and non-young breast cancer

The risk of young breast cancer (age of onset of breast cancer <35 years) was not significantly correlated with FPG. For the population with non-young breast cancer (>=35 years), the risk of breast cancer was significantly increased in the Q2 and Q3 groups compared with the Q1 group, with HR values of 1.21 (95% CI: 0.92–1.59) and 1.28 (95% CI: 0.98–1.67, respectively; see **Table 4** for details).

**Table 4:** Multivariate Cox regression analysis of the effect of FPG on the risk of early-onset and late-onset breast cancer.

Groups	Model 1 (age)		Model 2 (Others)	
	HR rate (95% CI)	P rate	HR rate (95% CI)	P rate
Early onset breast cancer				
Q1	1		1	
Q2	0.92 (0.33, 2.55)	0.88	0.90 (0.33, 2.50)	0.84
Q3	1.04 (0.31, 3.46)	0.95	0.96 (0.28, 3.27)	0.94
	P for trend = 0.99		P for trend = 0.91	
For every 1 mmol/L increase in fasting blood glucose	0.98 (0.49, 1.95)	0.95	0.92 (0.46, 1.87)	0.83
For every 1 standard deviation increase in fasting blood glucose^a^	0.99 (0.64, 1.52)	0.95	0.95 (0.61, 1.48)	0.83
Late onset breast cancer				
Q1	1		1	
Q2	1.22 (0.93, 1.59)	0.16	1.21 (0.92, 1.59)	0.17
Q3	1.30 (1.00, 1.69)	0.05	1.28 (0.98, 1.67)	0.07
	P for trend = 0.05		P for trend = 0.07	
For every 1 mmol/L increase in fasting blood glucose	1.03 (0.97, 1.09)	0.29	1.02 (0.97, 1.09)	0.43
For every 1 standard deviation increase in fasting blood glucose^b^	1.05 (0.96, 1.16)	0.29	1.04 (0.94, 1.15)	0.43

Model 1 corrected for age; model 2 corrected for systolic blood pressure, waist-hip ratio (<0.8 or ≥0.8), triglycerides, total cholesterol, frequency of physical exercise (infrequent or frequent), smoking (yes or no), alcohol consumption (yes or no), and salt intake (light or moderate). ^a^0.63, ^b^1.68.

### 3.5 Sensitivity analysis: multivariate Cox regression analysis of the effect of TyG index on disease risk in breast cancer

Compared with the W1 group, the HRs for the W2 and W3 groups were 1.19 (95% CI: 0.91–1.55) and 1.21 (95% CI: 0.91–1.60), respectively; see **Table 5**.

**Table 5:** Multivariate Cox regression analysis of the effect of TyG index on disease risk in breast cancer.

Groups	Model 1 (age)		Model 2 (Others)	
	HR rate (95% CI)	P rate	HR rate (95% CI)	P rate
Breast cancer disease				
W1	1		1	
W2	1.19 (0.91,1.55)	0.21	1.19 (0.91,1.55)	0.21
W3	1.20 (0.91,1.57)	0.19	1.21 (0.91,1.60)	0.19
	P for trend=0.21		P for trend=0.21	
For every 1 mmol/L increase in TyG indexC	1.14 (0.98,1.33)	0.1	1.15 (0.98,1.36)	0.09
For every 1 mmol/L increase in TyG index^d^	1.09 (0.98,1.21)	0.1	1.10 (0.99,1.23)	0.09

Model 1 corrected for age; model 2 corrected for systolic blood pressure, waist-hip ratio (<0.8 or ≥0.8), triglycerides, total cholesterol, frequency of physical exercise (infrequent or frequent), smoking (yes or no), alcohol consumption (yes or no), and salt intake (light or moderate). ^d^0.67.

## 4 Discussion

According to the latest statistics from the World Health Organization, breast cancer has become the most frequent malignant tumor in the world (14), which seriously damages the physical and mental health of women and places a great burden due to its social and economic aspects. Therefore, the exploration of risk factors for breast cancer and secondary prevention strategies for early intervention have received increasing attention. The latest research has shown that FPG level is closely related to the onset and prognosis of breast cancer. In our study, women with healthy physical examinations were classified according to FPG values. After follow-up, it was found that the incidence and density of breast cancer were positively associated with FPG levels. Multivariate analysis showed that elevated FPG was a risk factor for breast cancer. At the molecular level, hyperglycemia glycosylates protein structures non-enzymatically and promotes the production of various factors which affect the growth of tumor cells. Hyperglycemia can also activate the polyol pathway by increasing the expression and activity of aldose reductase, which subsequently increases the metabolic activity of breast cancer cells. In the process of catabolism, sugar produces reactive oxygen clusters such as superoxide anions which aggravate the oxygen stress in tumor cells, causing the obstruction or disorder of cell DNA and enzyme synthesis, thus inducing carcinogenesis and promoting the occurrence and development of breast cancer through a variety of pathways. In breast cancer cells, insulin-like growth factor (IGF)-I and IGF-II exert biological activity mainly through the IGF-1 receptor. After ligand and receptor binding, the IGFs exert antiproliferation and pro-phosphorylation effects on specific binding proteins and reduce their binding to the IGF-I receptor, thus promoting breast cancer development. It is well known that the specific components of the IGF system are ubiquitous, and the interference or disruption of any link in this system may cause growth retardation, atherosclerosis, insulin resistance and even cancer. Influencing mechanisms may involve insulin, IGF-1, insulin resistance (15, 16), endogenous hormones (17), leptin (18), adiponectin (19), inflammatory factors and other factors. Moreover, both Chinese and international researchers have found that FPG level is closely related to the efficacy of breast cancer and systemic therapy, metastasis and death risk (20–23). Nevertheless, the long-term impact of FPG on breast cancer risk and the specific biological mechanism need further analysis.

We divided the breast cancer study population into young and non-young breast cancer cohorts according to the age of onset, then performed Cox regression analysis based on FPG levels. The results suggest that FPG levels are not a risk factor for young breast cancer, which may be due to the relatively small population base in the young group, leading to some bias. The psychological neglect of malignant tumor diseases in young people may also reduce the diagnosis rate. Breast cancer is also associated with individual differences in diet and exercise habits and family history (24, 25). For patients with non-young breast cancer, FPG levels are a high-risk factor for morbidity, and for every 1 mmol/L increase in FPG, the risk increases (26). The average age of abnormal glucose metabolism is between 40 and 50 years (27), while the average age of early type II diabetes is around 33 (28), and studies have shown that the FPG level increases the risk of breast cancer (29). We can therefore conclude that routine testing of FPG in women aged 35 to 50 may be helpful for screening and early diagnosis and treatment of breast cancer.

The TyG index takes the logarithm of FPG and lipid indices and conducts multivariate analysis for the risk of breast cancer. Many domestic and foreign studies have shown that an elevated TyG index is independently associated with the increased risk of diabetes and cardiovascular disease in adults, indicating that it may be a reliable predictor of diabetes in high-risk groups (30–32). However, the results of the present study showed that the TyG index is not a risk factor for breast cancer, while FPG is a risk factor for the cumulative incidence of breast cancer. Therefore, we can infer that while blood lipids may affect the prognosis of breast cancer disease (33), they are not a risk factor for breast cancer development (34).

In conclusion, we found an increased cumulative incidence of breast cancer disease in people with high FPG but no significant difference in the effect of different FPG levels on the risk of young breast cancer among the population. It is rather a risk factor for non-young breast cancer.

## Data availability statement

The original contributions presented in the study are included in the article/supplementary material. Further inquiries can be directed to the corresponding authors.

## Ethics statement

The studies involving humans were approved by Kailuan LC Hospital Ethics Committee. The studies were conducted in accordance with the local legislation and institutional requirements. The participants provided their written informed consent to participate in this study.

## Author contributions

GL: Writing – original draft, Writing – review & editing. MY: Writing – original draft, Writing – review & editing. ZF: Writing – original draft, Writing – review & editing. FQ: Writing – original draft, Writing – review & editing.
